# The Faculty Development Paradox: Applying Competency-Based Education Principles to Faculty Development

**DOI:** 10.5334/pme.2603

**Published:** 2026-09-29

**Authors:** Teri Lee Turner, Jolanta Karpinski, Arvin K. Damodaran, Pedro Tanaka, Ming-Ka Chan, Severin Pinilla, Denyse Richardson, Mary Ellen J. Goldhamer, Linda Snell, Laura Edgar, As members of the International Competency-Based Health Professions Education Collaborators

**Affiliations:** 1 Department of Pediatrics, Baylor College of Medicine, Houston, Texas, USA; 2 University of Ottawa, Canada; 3 School of Clinical Medicine, Faculty of Medicine and Health, UNSW Sydney, Australia; 4 Stanford University School of Medicine, Stanford, California, USA; 5 Antiracism and Social Justice, Max Rady College of Medicine, Rady Faculty of Health Sciences, University of Manitoba, Winnipeg (Treaty 1 Territory and Homeland of the Red River Metis), Canada; 6 University Hospital of Old Age Psychiatry and Psychotherapy, University of Bern, Bern, Switzerland; 7 Division of PMR (Physiatry), Department of Medicine, University of Alberta, Edmonton, Alberta, Canada; 8 Division of General Internal Medicine, Massachusetts General Hospital, USA; 9 Harvard Kidney Urology Hematology Training Institute, Boston, Massachusetts, USA; 10 McGill University, Montreal, Canada; 11 Royal College of Physicians and Surgeons of Canada, Ottawa, Canada; 12 Competencies, Milestones, and Faculty Development, Accreditation Council for Graduate Medical Education, Chicago, Illinois, USA

## Abstract

Competency-based health professions education (CBHPE) has transformed learner education through explicit competencies, sequenced progression, direct observation, coaching, and programmatic assessment. However, faculty development—the mechanism that should prepare educators to enact CBHPE—has not undergone a parallel evolution. This misalignment limits the effectiveness and sustainability of CBHPE. In this paper, the authors propose a model of Competency-Based Faculty Development (CBFD) that applies CBHPE design principles to the growth of educators and introduces the Master Adaptive Teacher (MAT) as a developmental outcome for faculty. Using Van Melle’s five core components of CBHPE as an organizing framework, the authors synthesized existing educator competency frameworks, literature on adaptive expertise, faculty development theory, and implementation science and mapped these components to faculty development structures, strategies, and assessment processes. The core components can be directly translated to faculty development. Implementing CBFD requires (1) defining educator competencies aligned with institutional roles, (2) building developmental pathways linked to outcomes, (3) integrating flexible, workplace-based learning and coaching, (4) designing instruction that models CBHPE principles, and (5) adopting a longitudinal assessment program using multiple data sources. The MAT framework further operationalizes adaptive expertise as a core capability developed through reflection, coaching, community, and system-level support. In summary, CBFD represents a structural redesign that positions faculty as learners within a developmental ecosystem. When aligned with culture and institutional infrastructure, CBFD strengthens educator identity, enhances teaching quality, and enables CBHPE to achieve its full potential.

## Introduction

Competency-based medical education (CBME) has radically reshaped health professions education—introducing explicit competencies, progressive development, direct observation, feedback, coaching, and programmatic assessment to support learner growth and professional identity formation [1,2,3]. CBME has replaced vague benchmarks with structured roadmaps, transparent criteria for success, and systematic processes for assessment and feedback that guide continuous development [4]. Yet, in stark contrast, faculty development, the very mechanism intended to prepare educators for this transformation, remains unchanged. This paradox raises a critical question: if competency-based education has transformed the learner experience, why has it failed to similarly influence and inform faculty education and development? Without a parallel shift in faculty development, the promise of CBME risks being undermined, as educators may lack the structures and support required to deliver on its principles. This paper addresses that gap by making the case for competency-based faculty development (CBFD) and outlining practical strategies to align faculty growth with competency-based educational principles. In doing so, we recognize that faculty occupy dual roles as both learners and educators within CBME. Thus, competency-based faculty development must not only support faculty learning but also strengthen the teaching, coaching, feedback, and assessment practices through which competency-based education is enacted in daily educational work.

We argue that the same principles that underpin CBME for learners should equally shape faculty development. Van Melle and colleagues have articulated core competency-based educational components that guide design and implementation; we draw on these components and apply them directly to faculty development to move from paradox to practice [4]. Because these principles extend beyond medicine, we use Competency-Based Health Professions Education (CBHPE) to refer to the broader landscape. For this paper, we adopt Steinert’s 2024 definition of faculty development: *“Faculty development refers to all activities health professionals pursue to improve their knowledge, skills, and behaviors as teachers and educators, leaders and managers, and researchers and scholars, in both individual and group settings. It also refers to those programs and activities that are designed to promote change at the organizational level and influence the institutional culture in which faculty members work*.” Positioned from this definition, we intend to offer a reframing and practical pathways for competency-based faculty development (CBFD) [5]. Having outlined the rationale and scope of this paper, we now turn to the case for competency-based faculty development and its essential role in the success of CBHPE.

## Why Competency-Based Faculty Development Matters

CBHPE can only reach its full potential when faculty are prepared and supported to enact its principles. Faculty development is not peripheral—it is the structural backbone that enables educators to deliver on CBHPE’s promise. The case for CBFD starts with recognizing the profound shift in how the educator role has evolved under CBHPE. While content knowledge was historically the sole prerequisite for teaching, the implementation of CBHPE has yielded teaching competency frameworks that outline faculty roles and expectations well beyond traditional instruction [6,7]. This shift has made faculty development essential, yet it creates a developmental gap: experienced clinician educators, often trained under an input based paradigm (e.g., lectures and time), may feel disconnected from contemporary CBHPE, which is anchored to demonstrating outcomes. The difference represents a crucial opportunity. Rather than viewing this as a deficit, CBFD reframes the challenge as a chance for educators to personally experience and navigate the very same outcome-based, competence focused educational paradigm to which they are now expected to contribute [8]. This direct, experiential learning would enhance their understanding of the challenges and benefits of current learning models, ultimately improving their ability to coach and support their own learners and to enact key teaching practices in CBHPE, including coaching, feedback, workplace-based assessment, and learner supervision.

This experiential approach also catalyzes a deeper, broader rethinking of how faculty development is designed and delivered. Grounded in constructivist principles, CBFD offers more than skill-building—it signals a cultural shift. Faculty development becomes an agent of culture change, promoting growth-oriented mindsets and safe learning environments where vulnerability and lifelong learning are normalized [9]. Framing development as empowering rather than evaluative reduces resistance and inefficiencies, aligning faculty growth with institutional goals.

At the same time, cultural change must respond to structural realities. The transition to CBHPE has fundamentally expanded the scope and complexity of the educator role. The traditional role of the subject matter expert or information provider has evolved into a multitude of specialized roles essential for CBHPE to function effectively [7]. This expansion has resulted in a larger and more functionally diverse group of educators. Consequently, faculty development can no longer rely on a uniform, episodic, workshop-based approach. To effectively support the entire CBHPE system, faculty development must evolve to address the unique, individualized developmental needs of each of these varied educator roles. This focused and tailored approach is essential to ensure every faculty member, regardless of their specific title or discipline, possesses the necessary skills and confidence to execute their distinct function within CBHPE, thereby supporting learner progression and supporting overall program quality. Taken together, these realities underscore that CBFD is a structural necessity for CBHPE. Advancing CBFD will require cultural and system alignment, including expectations, protected time, coaching models, and accountability mechanisms, to support faculty growth within a competency-based system [5]. Without this parallel shift in faculty development, institutions risk undermining CBHPE’s goals, leaving educators unprepared and learners underserved.

## Reflexivity and Positionality Statement

Before outlining our approach, we acknowledge our positionality. As authors based in the Global North, our perspectives on CBHPE and CBFD are shaped by professional roles in well-resourced academic institutions and years of implementing CBHPE reforms in these settings. Our assumptions and interpretations are therefore grounded in systems with structural supports, accreditation standards, and educational cultures that may differ markedly across regions. This positionality includes privileged access to resources, policy influence, and established CBHPE infrastructure, shaping how we understand implementation challenges and opportunities. We also recognize that frameworks developed in the Global North may not fully reflect local needs, values, or constraints elsewhere, and we aim to engage with humility, openness, and respect for diverse approaches. Accordingly, we have sought to remain reflective about how our positions shape our recommendations and to emphasize principles intended to be culturally and contextually adaptable.

## Applying Van Melle’s Five Core Components to Faculty Development

Having outlined the rationale for CBFD and situated our perspective, we now turn to practical strategies for operationalizing CBFD. Van Melle et al. (2019) published a framework for evaluating CBHPE programs, describing five core components: outcome competencies, sequenced progression, tailored learning experiences, competency-focused instruction, and programmatic assessment [4]. We propose that these components are also applicable to faculty development and can guide the adaptation of CBFD for educators. Table 1 presents a competency-based framework for faculty development systems grounded in Van Melle’s five core components. Table 2 translates this framework into practice by offering actionable strategies aligned with each component to support implementation and individual educator growth over time.

**Table 1:** Applying Van Melle’s Five Core Components of CBHPE to Faculty Development (FD) Systems.


CORE COMPONENTS OF CBHPE					

CORE COMPONENT	OUTCOME COMPETENCIES	SEQUENCED PROGRESSIVELY	TAILORED LEARNING EXPERIENCES	COMPETENCY-FOCUSED INSTRUCTION	PROGRAMMATIC ASSESSMENT

**Definition** [10]	Competencies required for practice are clearly articulated (and are identified based on societal needs).	Competencies and their developmental markers are sequenced progressively (in a way that supports a learner’s progression from novice to master).	Learning experiences (that resemble the practice environment) facilitate the development of competencies (and are flexible enough to accommodate variation in individual rates of progression).	Teaching practices promote the developmental acquisition of competencies (and emphasize learning through experience and application as well as knowledge acquisition).	Assessment practices support and document the developmental acquisition of competencies (and emphasize workplace-based observation as well as meaningful feedback).

**Proposed Faculty Development Program components**	EPAs for Teachers or Entrustable Teaching Activities (ETAs) [11,12] Fundamental Teaching Activities [13] Clinician Educator Milestone Domains [14] Academy of Medical Educators Professional Standards’ Domains for medical dental and veterinary educators [15] Teacher Role-specific competencies [16,17]	Clinician Educator Milestones Mixed-experience community of practice (novice, mid-career, senior) to support progression Professional Identity formation as an educator Longitudinal development [16]	Workplace-based learning (peer coaching to improve) Just-in-time faculty development Use existing group meetings for teacher development Individualized Needs Assessments and Development Plans as a Teacher	Master Adaptive Learner cycle as an instructional backbone: Plan–Learn–Assess–Adjust Coaching (includes peer coaching) Deliberate practice Desirable difficulty/productive struggle Scaffolding Mindset shifts from fixed to growth Competency-focused remediation using targeted instruction, coached practice, and reassessment to achieve defined educator competencies	Systems lens towards faculty development Longitudinal educator portfolios that go beyond trainee evaluations [18,19] Data collection metrics to score teacher feedback comments [63] Peer observation and feedback Planned, periodic structured observations of teaching (e.g., OSTEs or comparable observed teaching events) used as developmental checkpoints [20] Dashboards to monitor program-level trends in educator development


**Table 2:** Proposed Actions for Implementing a Faculty Development (FD) Program in Alignment with Van Melle’s Five Core Components of CBHPE.


DEFINING ACHIEVEMENT	MAPPING DEVELOPMENT	MEETING DIVERSE NEEDS	MODELING WHAT WE TEACH	EVALUATING GROWTH

Select a competency framework appropriate for your context (e.g., EPAs for Teachers, CE Domains from CE Milestones, or role-specific competencies). Map existing FD activities to the framework and identify gaps in offerings. Align outcome competencies with program/institutional goals to ensure strategic relevance. Use outcome competencies to create job descriptions and role expectations. Develop digital badges or micro-credentials linked to specific competencies. Design faculty teaching assessments that reflect outcome competencies.	Label FD offerings by developmental level (e.g., Teaching 101: Foundations of Clinical Coaching, Teaching 201: Advanced Feedback & Remediation). Publish learning pathways (e.g., New Preceptor → Coach → Educational Leader). List prerequisites or “recommended before” experiences so faculty can self-select. Design communities of practice with mixed-experience (novice, mid-career, senior educators together) around key roles (e.g., simulation, small-group teaching). Pair novice and more experienced educators for observation Create developmentally scaffolded failure-sharing sessions in which senior educators model learning-from-failure to support identity formation and normalize growth over time.	Have faculty complete a brief competency-mapped self-assessment to identify 1–2 priority growth areas for the next quarter. Have faculty gather 360-degree input (learners, peers, supervisor/education leader) using a short, structured tool tied to the same competencies. Use asynchronous modules to build foundational knowledge at self-selected pace, then apply learning in future teaching sessions. Use peer- or AI-supported practice to rehearse feedback, questioning, or coaching by simulating learner responses before a real encounter. Use AI to create just-in-time supports (e.g., checklist, flashcards, 5-question quiz) from a key article or guideline for an upcoming teaching moment. Establish a standardized peer observation-and-debrief process using competency-aligned checklists to support coached improvement.	Design FD sessions around explicit educator competencies and replace ‘objectives’ with ‘competency’ aims. Build expert modeling into sessions (live or video) so faculty can observe competent practice. Design skills labs (e.g., feedback lab, coaching lab) with multiple short practice cycles and coaching between attempts. Break complex educator skills into component parts (i.e., needs assessment, writing goals and objectives, instructional design) and teach them sequentially. Design sessions so faculty experience scaffolding themselves, reinforcing how to use it with learners. Replace ‘send to a workshop’ or ‘do a module’ remediation plans with coached, observed, practice-based sessions. Normalize practice, rehearsal, and struggle in FD sessions and that teaching is a skill that can be developed, not a trait.	Hold an annual FD review meeting that examines aggregated data to identify program-level strengths and growth priorities. Implement a standardized educator portfolio template that includes selected observation feedback, reflections on growth edges, and development goals over time along with learner and peer observer feedback and evaluations. Periodically review a small sample of narrative feedback comments from learner evaluations using a brief rubric to assess specificity and actionability. Launch a voluntary peer observation process using a shared competency-based template. Incorporate a standardized OSTE as a periodic sampling tool and use aggregated findings to inform FD priorities, resource allocation, and program redesign. Create a competency heat map to visualize patterns in educator development and identify areas for targeted FD investment.


### Outcome Competencies: Defining What Faculty Should Achieve

Outcome competencies for faculty development encompass the abilities required to fulfill the roles of the health profession teacher or the health profession educator. A range of frameworks for educators, expressed as competencies, Milestones, fundamental tasks, and entrustable professional activities (EPAs) (i.e., Yilmaz et al., 2023; van Bruggen et al., 2021; Dewey et al., 2017; Accreditation Council Graduate Medical Education Clinician Educator Milestones 2022; Academy of Medical Educators Professional Standards 2021; Srinivasan et al., 2011; Molenaar et al., 2009) have been published [7,11,12,14,15,17,21]. These frameworks include a diverse set of competencies, such as those applicable to teaching in classroom and clinical settings, as well as those applicable to leaders and administrators of educational programs. Consequently, the relevance of these outcome competencies will vary among teachers and educational settings. For example, competencies required in the undergraduate medical education classroom differ from those required in clinical or laboratory settings, and frameworks derived in North American and European settings may be irrelevant or omit the competencies required of teachers in the Global South. CBFD programs will need to carefully consider the educational context, role of the teacher/educator, and purpose of the competency framework to determine the outcome competencies relevant to their context.

To begin using outcome competencies, start by selecting an outcome competency framework (see Tables 1 and 2) through a small advisory group that explicitly includes learners, the recipients of teaching. Next, make competencies visible in real educator roles by embedding them in position descriptions (e.g., “clinical preceptors provide feedback, coaching, and supporting clinical reasoning/decision-making”) and publishing a concise role-to-competency map (coach, small-group facilitator, simulation educator, course or rotation leader, assessment lead) [16,17]. We propose aligning faculty development to the framework by tagging offerings to specific competencies and recognizing progress with digital badges or micro-credentials linked to demonstrated competence. This approach turns outcome competencies from statements on paper into everyday guides for growth. Once the framework is set, reinforce role-aligned expectations through onboarding and annual development conversations. When educator roles are articulated this way, individuals across the health professions can see themselves in the framework and quickly identify which competencies matter most for their current practice and career stage.

### Sequenced Progression: Mapping Development Over Time

In CBFD, a sequenced progression of competencies provides an organizational structure for faculty development programming by identifying prerequisite abilities and describing subsequent levels for skill attainment. Several existing competency frameworks incorporate sequenced progression. The Accreditation Council for Graduate Medical Education (ACGME) Clinician Educator Milestones outline a developmental progression from novice to expert, and Table 3 provides a sample individual faculty development plan that uses the Clinician Educator Milestones to operationalize this progression over time [14]. The Canadian College of Family Physicians framework of fundamental teaching activities delineates key roles for teachers (e.g., clinical preceptor, educational leader) and outlines different expectations as teachers progress from applying basic educational principles to demonstrating leadership or scholarship in the same activity or role [13].

**Table 3:** Sample Individual and Program-Level Competency-Based Faculty Development Plans.


SAMPLE INDIVIDUAL FACULTY DEVELOPMENT PLAN USING THE PRINCIPLES OF CBHPE	SAMPLE FACULTY DEVELOPMENT PLAN AT THE PROGRAM LEVEL USING THE PRINCIPLES OF CBHPE

***An example of an early-career faculty member using ACGME Clinician Educator Milestone “Educational Theory and Practice 11: Curriculum Development”*** [14]: Identifies roles and responsibilities as a faculty member. Reads the CEM Domains of Competence (CCF – Outcome Competencies). Performs a self-assessment using the CEM (CCF – Sequenced progressively). Asks trusted peer-faculty to observe bedside teaching and provide a CEM assessment on the subcompetency (Level 1). Creates a goal and identifies steps necessary to improve in the subcompetency (Achieve Level 3 within 1 year) (CCF – Tailored learning experiences); can be done independently or with a peer/mentor. Begins to identify as an educator. Identifies and participates in specific self-learning and faculty development and asks for peer-coaching. Includes reading Curriculum Development for Medical Education: A Six-Step Approach and an online course on competency-based curriculum development. (CCF – Competency-focused instruction) Seeks opportunities to enhance experiences by asking the program director to assist in adapting the curriculum for a series of didactics identified by the PEC. After a period of learning and additional experiences, repeats process of self-assessment and peer observation, with input from peer coach. Faculty member now at a Level 3. Adds each completed activity to an Educator Portfolio (CCF – Programmatic assessment). Identifies opportunities to help other faculty who would like to begin learning and seek opportunities to lead a curriculum development project. Continues to work on the subcompetency to achieve mastery (Level 5). At any point in this process, identifies a second CEM subcompetency to assess and work on.	Define explicit faculty outcome competencies aligned with local teaching roles (e.g., clinical supervisors, advisors, educational leader). Consider introducing teaching EPAs and developing entrustment decisions analogous to trainee EPAs. Map faculty roles to sequenced developmental educator milestones. Use frameworks such as the Accreditation Council for Graduate Medical Education Clinician Educator Milestones or another educator competency framework. Design a faculty development curriculum from novice to expert, including: tailored, flexible learning experiences (e.g., online micro-modules and specialty-specific resources), faculty coaching infrastructure with trained educational coaches or peer coaching available for longitudinal guidance, Individualized faculty learning plans informed by self-assessment, observation, learner/peer/coach feedback, and CEM. Structured reflection prompts, learning portfolios, and guided self-assessment. Design programmatic assessment of teaching competence with multiple data points including: learner feedback and peer review, OSTEs and teaching artifacts (e.g., course blueprints, presentations, instructional materials), coaching plan, consider a Teaching Competence Committee to review progress and advise on development plans at the program level. Establish communities of practice or learning communities to support shared learning, identity development, and collaborative reflection. Ensure protected time and recognition for participation in longitudinal faculty development activities, including consideration in promotion criteria. Use developmental quality frameworks (e.g., Association for Medical Education in Europe [AMEE] ASPIRE criteria; amee.org) to evaluate and improve the program’s faculty development systems. Promote institutional culture of innovative teaching and faculty development. Communication with an annual “Teaching Expectations Letter” (analogous to an APE) to clarify expectations, goals, and required competencies for clinician educators, Foster growth mindset, psychological safety, and opportunities for “productive struggle” and innovation in teaching.


**Legend:** APE: Annual Program Evaluation, CBFD: Competency-based faculty development, CBHPE: Competency-Based Health Professions Education, CCF: Core components framework, CE: Clinician Educator, CEM: Clinician Educator Milestones, PEC: Program evaluation committee

Throughout a teacher’s career, the sequenced progression of CBFD can reduce redundant training and help educators build on their existing skill base as they modify roles, develop professional identity, and adapt to changing curricular expectations. Programs can operationalize this progression by labeling offerings as 101–201–301 levels similar to college courses, structuring mixed-experience communities of practice, and pairing novice educators with more experienced peers for observation and coaching. Intentionally creating spaces where senior educators share failures as well as successes further supports professional identity formation and normalizes growth over time. Table 3 also includes a parallel program-level example that demonstrates how sequenced progression can be built into a coherent faculty development strategy. The challenge for faculty development is to design, describe, and market programming aligned with the sequenced progression of competencies.

### Tailored Learning Experiences: Meeting Diverse Faculty Needs

In CBFD, tailored learning experiences rely on identifying a teacher’s learning needs and ensuring that the faculty development program is flexible enough to meet them. An individual teacher’s needs will vary based on demonstrated competencies, prior experiences, current teaching scope, and future career goals. Learning needs can be identified through brief competency-mapped self-assessments complemented by 360-degree input from learners, peers, and educational leaders. Clear descriptions of the competencies required for different teaching settings and roles can further support educators in articulating and prioritizing their learning needs [17,21].

Faculty development has often been synonymous with group-based, inflexible formats (e.g., workshops, seminars, courses). In contrast, tailored approaches can include asynchronous online modules, peer coaching, and other flexible learning options that allow educators to individualize both timing and content [5]. For example, a pharmacist educator transitioning into a small-group facilitator role might complete a short online module on group dynamics, participate in peer coaching with an experienced facilitator, and use guided reflection to refine their approach before leading sessions independently.

In addition, Artificial Intelligence (AI) may support tailored learning through safe rehearsal and just-in-time resource development. Educators can use an AI assistant to simulate learner responses while practicing feedback conversations, questioning strategies, or coaching approaches, iterating until wording and sequencing feel natural for workplace use. AI-enabled content creation can also generate checklists, brief quizzes, or flashcards from key resources to support an upcoming teaching encounter.

### Competency-focused Instruction: Modeling What We Teach

Competency-focused instruction provides opportunities to learn and practice new skills in settings that mirror authentic teaching practice, allowing context-specific learning to be embedded in real-world environments. Historically, faculty development programming has focused on skill application, albeit in group-based learning activities (e.g., feedback workshops). In contrast, Steinert (2010) has called for professional development to be situated in the workplace, and CBFD further advances this by intentionally designing instruction that incorporates coaching, deliberate practice, rapid-cycle practice with debriefing, scaffolding, and productive struggle [22]. In doing so, CBFD cultivates adaptive expertise by engaging educators in the Master Adaptive Learner cycle—practice, feedback, reflection, and adjustment—within authentic teaching contexts [23]. Within this model, faculty development sessions are structured around explicit educator competencies rather than traditional objectives, with expert modeling used to make competent practice visible and multiple short practice cycles to support skill acquisition. Complex educator skills can be broken into component parts and taught sequentially, allowing faculty to experience scaffolding firsthand and reinforcing how to apply these strategies with learners.

In CBFD, competency-focused instruction should align learning, assessment, and faculty support with institutional goals. Robertson and colleagues demonstrated the feasibility of institutional implementation through competency frameworks that align faculty development, recognition, and promotion systems [24]. Importantly, competency-focused instructional design has direct implications for faculty remediation: rather than relying on “send to a workshop” or “complete a module” approaches, CBFD emphasizes coached, observed, practice-based experiences to address specific competency growth edges. By normalizing practice, rehearsal, and productive struggle—and by explicitly promoting a growth mindset—competency-focused instruction reinforces the principle that teaching is a skill set that can be developed over time, not a fixed trait [9].

### Programmatic Assessment: Evaluating Faculty Growth Holistically

Applying the CBHPE principles of programmatic assessment requires a systematic approach to designing and implementing assessment practices that support and document teachers’ development and demonstration of competence over time. A program of assessment should draw on multiple assessment methods and sample across the range of desired outcome competencies, with an emphasis on low-stakes, longitudinal evidence rather than single-point judgments. Learner feedback remains the most used method for assessing teaching performance, but it is insufficient on its own and should be complemented by additional approaches [20]. Objective structured teaching encounters (OSTEs), for example, have been developed for a range of teaching scenarios and can function as planned, periodic checkpoints that provide comparable evidence of growth when used longitudinally rather than as stand-alone evaluations. Similarly, proposed EPAs for teachers offer broad coverage of university teaching activities; however, international expert consultation emphasized the importance of tailoring assessment frameworks to local contexts and priorities [11].

CBFD program designers must therefore select assessment methods that are aligned with desired outcome competencies and embedded within a coherent program of assessment. This includes making intentional decisions about how assessment data are used to support both assessment *for* learning—through feedback, coaching, and identification of growth edges—and assessment *of* learning, such as informing recognition or role progression. A programmatic assessment approach shifts the focus from evaluating individual data points to synthesizing patterns across time and sources, often supported by program-level tools such as educator portfolios, aggregated dashboards, or unit-level competency heat maps that allow faculty development leaders to monitor trends and guide program improvement.

The CBHPE approach to faculty development, however, cannot flourish in an environment ill-suited to its growth. Distinct from but related to the learning environment, the teaching environment reflects an organization’s systems of reward and recognition, institutional resources, communities of practice, and prevailing beliefs about teaching and professional identity. The growth mindset and developmental orientation required by CBHPE depend on psychological safety, relational trust, and a culture that normalizes observation, feedback, and coaching [25,26]. CBFD must therefore extend beyond individual programs or assessment tools to include intentional organizational change efforts that align culture, incentives, and structures with the goal of continuous educator development [5].

While the five components offer a clear blueprint for CBFD design, they do not, on their own, specify the developmental outcome for faculty or the mechanisms that sustain growth over time. The Master Adaptive Teacher (MAT) framework helps connect CBFD’s design features to the adaptive expertise faculty need in practice [27].

## From Components to Capability: The Master Adaptive Teacher Framework

Master adaptive teachers do not simply apply educational strategies consistently; they modify their approaches in response to learner needs, context, performance data, and evolving educational environments. For example, they may adjust feedback strategies based on learner readiness, alter supervision levels as competence develops, or redesign teaching approaches in response to curricular or system changes. Building on this, CBFD does more than teach discrete skills; it creates the conditions for adaptability and evolution over time. Because clinical teaching occurs in complex, changing environments, CBFD must support faculty in developing adaptive expertise through reflection, responsiveness, and flexibility. Faculty occupy dual roles as both learners and educators; therefore, the ultimate test of CBFD is not simply what faculty learn, but how that learning is translated into teaching, coaching, assessment, and supervision practices that support the development of others. To operationalize this goal, we introduce the Master Adaptive Teacher (MAT) framework for health professions educators, adapted from the Master Adaptive Clinician Educator (MACE) framework in medicine [27]. CBFD advances MAT by embedding these qualities into faculty development processes, modeling adaptive expertise, fostering growth-oriented mindsets, and providing longitudinal coaching, thus making MAT not just an aspiration, but actionable. Examples of how these principles can be translated into individualized and program-level faculty development plans are shown in Table 4.

**Table 4:** Institutional Strategies to Support Master Adaptive Teacher (MAT) Development within Competency-Based Faculty Development (CBFD).


DOMAIN	CORE PURPOSE	KEY FEATURES/MECHANISMS

**Coaching & Feedback**	Make adaptive growth explicit and actionable	Coaching is crucial for making learning effective. Mentors and supervisors should use a growth mindset coaching approach, offering tools for providing immediate, actionable feedback. The process of guided self-reflection using ACGME Clinician Educator Milestones can lead to an Individualized Plan for professional development. Effective feedback must be specific and actionable, provided within a trusting educational alliance [23,28].

**Identity Formation & Learning Environment**	Support educator identity and tolerance of complexity	Faculty development programs, particularly those designed as longitudinal curricula with ongoing community of practice support, are effective in enhancing teaching confidence and supporting educator identity formation [29,30]. The learning environment for faculty must be intentionally supportive, fostering a culture of tolerance of ambiguity and providing space for “productive struggle” and reflection on failure [31,32].

**Empowerment & Autonomy**	Enable intentional adaptability and innovation	Adaptive educators demonstrate a commitment to supporting learner and teacher autonomy and encouraging calculated, beneficial risk-taking, known as “beautiful risks”. Empowerment is fostered through institutional and program commitment to faculty development, allocation of resources, and recognition of teaching excellence [33,34].

**Community of Practice**	Sustain development through shared learning	Establishing communities of practice enables faculty to share experiences, challenges, and strategies for growth. By fostering trust and psychological safety, these networks normalize reflection, feedback seeking, peer observation, and collaborative problem solving. Such communities support ongoing professional development while creating conditions for adaptive learning and continuous improvement [22,35].

**System-Level Alignment**	Ensure sustainability and legitimacy	Institutions must align incentives, recognition, and career advancement structures with the goals of competency-based faculty development. This process includes rewarding teaching excellence, providing protected time for professional development, integrating faculty development outcomes into promotion criteria, and building a compelling case for institutional investment by demonstrating value through educator effectiveness, workforce retention, and educational innovation [5,36].

**Resources & Infrastructure**	Maximize access, efficiency, and value of resources and infrastructure to support change [65].	Reduce unnecessary costs by utilizing and sharing existing resources (e.g., R2C2 coaching materials [64], podcasts based on existing materials), leveraging the strengths of professional societies (e.g., speaker bureaus), and optimizing virtual learning. Although faculty development requires investment, organizations may realize long-term value through reduced burnout and turnover and by developing MATs who can lead future curricular reform.


CBHPE has reshaped health professions education, requiring faculty to move beyond routine teaching toward adaptive expertise [33,37]. The MAT in HPE represents this future educator: a leader who continually develops skills, innovates, and reflects in order to adapt over time [27]. This concept parallels the Master Adaptive Learner (MAL) model, which emphasizes lifelong learning and adaptability [38]. Adaptive expertise balances efficiency with innovation to address unfamiliar challenges [37,39].

Becoming a MAT transforms professional identity through “professional coherence”, aligning actions with core values [33]. Essential traits for the MAT include curiosity, motivation, growth mindset, resilience, and interpersonal qualities such as honesty and humility, which promote psychological safety for learners [31,32,37]. MATs adopt “intentional adaptability,” a commitment to adapting to diversity and differing priorities across contexts, thereby proactively responding to changes in the clinical learning environment [40].

### Operationalizing Adaptive Expertise

To move from competency statements to adaptive performance, CBFD must specify how faculty learn to adapt. The Master Adaptive Learner (MAL) cycle—Planning, Learning, Assessing, and Adjusting—offers a process model for cultivating the reflective, iterative habits that underlie MAT development [27,38]. Strategies such as “intentional adaptation” and “weighted curation” (intentional selection, organization, and evaluation of professional activities where specific, high-priority areas are assigned higher values or greater significance than others) guide resource selection and evaluation [34]. CBFD must explicitly support these metacognitive processes, as reflective practice is critical for navigating complexity in clinical education [32,33].

MAT development is strengthened when these adaptive processes are anchored to established competency models, such as EPAs and Clinician Educator (CE) Milestones [41,42]. EPAs provide clinical context for integrating competencies. The ACGME CE Milestones clarify educator expectations developmentally from novice to expert [14]. These milestones encourage periodic self-assessment and reflection to identify proficiency and growth needs [28]. By integrating EPAs and milestones into faculty development programs, institutions can create structured pathways for educators to advance their skills, adapt to evolving educational demands, and achieve recognition for teaching expertise [41,42]. Table 3 provides recommendations and examples for embedding these frameworks into faculty development plans, while Table 4 outlines the essential institutional support required to implement and sustain this work. Realizing this vision requires sustained institutional commitment, including protected time, infrastructure, and financial support. While resource constraints may limit implementation in some contexts, particularly in under-resourced settings, opportunities exist to extend impact through adaptation of existing materials, collaboration with professional societies, shared resources, and scalable virtual approaches. These strategies should not be viewed as substitutes for investment, but as mechanisms to enhance the reach and sustainability of CBFD efforts. Framing faculty development as core educational infrastructure reinforces its role in advancing educational quality, faculty retention, and organizational adaptability. By aligning institutional commitment with the intentional development of adaptive educators, organizations can cultivate a cadre of Master Adaptive Teachers poised to lead health professions education into an increasingly complex future [27,31].

## Implementation of CBFD: Shifting the System

Implementing CBFD requires far more than expanding workshop offerings or adding new teaching checklists. CBFD represents a fundamental redesign of how institutions conceive of, support, and reward teaching [43]. It asks organizations to treat educator growth with the same clarity, rigor, and intentionality that CBHPE applies to learners [1,2,4,24]. And like the early evolution of CBHPE—when curriculum revisions proved insufficient without deeper shifts in culture, systems, and assessment structures—CBFD will falter if it is layered onto existing models without reexamining the assumptions that shape how faculty learn, improve, and are recognized [44,45]. Moving from traditional workshop-based approaches to a developmental, competency-aligned system requires rebuilding the underlying architecture of faculty development itself: the mindsets, structures, incentives, and relationships that enable educators to grow and improve across their careers. Importantly, CBFD is enacted within institutional culture, and what organizations truly value—made visible through promotion pathways, recognition practices, and day-to-day norms—will strongly shape what is prioritized, supported, and sustained.

A persistent barrier to implementation is misalignment between what CBFD seeks to develop and what academic systems most reliably reward. Academic systems continue to reward research productivity, clinical volume, and administrative efficiency more consistently than growth as an educator [5,46,47]. Faculty members often lack the time, recognition, and institutional support needed for meaningful engagement in developmental work [5]. Addressing this requires reexamining and realigning organizational levers, promotion pathways, workload expectations, recognition systems, and accountability structures, so faculty development becomes integral to professional identity rather than an optional add-on [5,36]. Leadership must articulate the rationale, provide visible support, and reinforce new practices with infrastructure, time, and resources rather than relying on individual goodwill.

Another challenge is that traditional faculty development activities often fail to align with desired outcomes. Accessible formats such as workshops and seminars, while common, rarely provide individualized, developmental experiences [8]. Formal longitudinal programs, which emerged in the 1990s, offer feedback, reflective practice, work-based learning, peer review, and communities of practice, but require significant resources [8]. Recently, modular, competency-based programs tailored to career stage have gained traction [48]. Yet, identifying outcome competencies is only one piece of the CBFD puzzle; effective implementation demands systemic support and alignment with institutional priorities.

Successful implementation also requires a fundamental mindset shift. CBFD depends on viewing teaching as a developmental competency rather than an inherent or static attribute [8,49]. Faculty and institutions must adopt a growth-oriented perspective that recognizes teaching expertise as something developed through deliberate practice, longitudinal feedback, observation, and supported progression [24,27]. This mindset transition shifts faculty development from check-the-box participation to continuous professional learning. Importantly, such transformation cannot be mandated. It must be nurtured within psychologically safe environments that invite participation rather than compel it. A supportive culture reduces stigma around observation and feedback and normalizes development as an expectation rather than an exception. Because mindset and identity work are central to sustained behavior change, asking faculty to adopt new practices without addressing beliefs about teaching competence and professional role identity is unlikely to produce lasting change [35].

Co-production with learners across the system, including trainees as recipients of teaching and faculty members as learners within CBFD, is essential to this shift. Learners bring unique perspectives on teaching behaviors and can contribute meaningfully through shared goal setting, collaborative teaching practices, structured upward feedback, and participation in coaching structures [50,51,52]. This approach moves beyond satisfaction surveys toward genuine developmental partnership, reinforcing shared responsibility for the learning environment [52,53,54]. Such bidirectional learning structures reflect the principles of a learning system, one in which improvement is distributed rather than hierarchical and faculty and learners develop together.

Implementation science offers the tools needed to translate CBFD from aspiration to sustained practice. An implementation lens reframes faculty development as a developmental ecosystem shaped by culture, context, and workflow, rather than a calendar of discrete events [5,24,55]. By diagnosing local conditions, identifying barriers and enablers, and tailoring strategies to institutional priorities and resources, implementation science helps explain why change succeeds or stalls and what is required to sustain new behaviors [56]. Core elements of CBFD—routine observation, structured feedback, sequenced developmental pathways, and longitudinal documentation of growth align naturally with implementation approaches that emphasize iterative testing, adaptation, and continuous improvement [57]. Frameworks such as the Consolidated Framework for Implementation Research (CFIR) further underscore the importance of organizational readiness, leadership engagement, and aligned incentives for system-level adoption [58].

Viewing implementation through a learning health system lens further strengthens CBFD. Deliberately developmental organizations (DDOs) offer an aspirational, but practical model in which growth is embedded in the daily fabric of organizational life rather than relegated to periodic workshops [59,60,61]. Evidence from graduate medical education demonstrates that coaching cultures grounded in DDO principles—developmental communities, shared aspirations, and consistent developmental practices normalize vulnerability, reframe failure as an opportunity for learning, and position growth as everyone’s responsibility [60,61]. When assessment data are used as fuel for development rather than judgment, organizations align incentives, foster psychological safety, and support ongoing improvement [62]. In this model, CBFD becomes embedded in routine practice, creating more consistent, equitable, and developmentally aligned teaching across the system. Implementation also requires mechanisms to evaluate impact at multiple levels—faculty behavior change, learner outcomes, learning environment measures, and institutional educational goals. The aim is not to build a better workshop calendar; it is to build a learning organization for educators.

Taken together, these elements—system alignment, cultural transformation, learner partnership, and intentional change leadership—form the foundation necessary for competency-based faculty development to thrive. Just as the opening moments of CBHPE required a shift from curriculum to culture, CBFD requires institutions to rethink how educator learning is supported, recognized, and sustained.

## Conclusion

CBHPE cannot thrive in a system where only learners are expected to develop. Its promise will not be realized unless faculty development evolves alongside it. CBFD can drive cultural change, not just fill gaps, by building systems that support and assess teaching competence, treating faculty as learners, and committing to continuous growth. The roadmap for CBFD is both possible and necessary. Institutions must build systems that support and assess teaching competence, treat faculty as learners, engage them in co-creation, and commit to continuous growth rather than one-time training. By doing so, faculty development will not only support individual educators but also drive institutional transformation, ensuring that both learners and teachers are equipped to meet the demands of modern health professions education.

## Disclaimer

The opinions expressed in this article are those of the authors and do not necessarily reflect American Medical Association policy or the views of other federal agencies.
